# Mr. Jukes's Cases of Ruptured Intestine

**Published:** 1818-04

**Authors:** Alfred Jukes


### Journal and Review.

VOL. V.] APRIL, 1818. [NO. 28.

### PART I.

### ORIGINAL COMMUNICATIONS.

——— quid utile ———

To Dr. PALMER.

Sir,

ALTHOUGH I am not aware that any practical inference can be drawn from a detail of the following cases of ruptured intestine; yet, as facts, I think that they are not altogether destitute of interest; and that, probably, you may consider them worthy of insertion into your Journal.

Two hasty sketches with the pen, of the portions of bowel removed, I have taken the liberty also of enclosing. They will give a tolerably correct idea of the nature and extent of the intestinal lesion; and, though perhaps not fit for etchings to be attached to a Journal,* may yet be deemed by you as sufficient memoranda.

I remain, Sir, with respect, yours, &c.

ALFRED JUKES.

Case 1. On Tuesday, November 6th, 1816, about two o'clock in the afternoon, William Loach, aged fourteen years, was brought to the hospital, labouring under the effects which resulted from the tread of a horse upon his

* The Etchings, illustrative of Mr. Jukes's Cases, will be given at the close of our present Volume. Editors.

belly. This occurrence had happened early in the morning of the preceding day; since which time, the poor fellow had been in excruciating pain, with frequent vomiting. I had him immediately taken to bed, and fomentations applied to his belly and extremities, which were exceedingly cold. His pulse was scarcely discernible, and on the countenance were depicted all the fatal consequences of peritonæal inflammation. His eyes were sunk, with a dark areola around them, exhibiting great anxiety. About an hour after his admission, some warm broth was given to him, and remained in his stomach about thirty minutes. An enema, administered in the course of the evening, afforded him but little relief. Thirty minims of tinct. opii, which he now swallowed, were instantly returned. Late in the evening, it was deemed adviseable to immerse him in a tepid bath, and afterwards to apply an emollient poultice over the whole abdomen. He seemed to experience some little ease from this treatment, and now and then appeared to slumber; but the frequent eructations of fluid into his mouth debarred all hopes of rest. In this state he continued until half-past two o'clock on the following day, with no alleviation of his symptoms, when he seemed quite wearied out, and expired.

*Dissection.* The omentum and peritonæum adhered firmly together, and were with difficulty separated. Coagulable lymph had formed a firm union, both as respects the abdominal parietes and the surface of intestine. Upon raising the omentum, fæculent matter freely issued from various openings through the coagulable lymph, which had completely embedded the smaller intestines with each other. The peritonæum, however, in some parts, was free from inflammation, as also portions of the bowels. The traces of it seemed principally confined to the vicinity of injury. On removal of the lymph, and tracing the course of the intestine, I at length discovered the ruptured portion; an etching of which I have attached to the Case.

CASE 2. Samuel Thornton, aged thirteen years, was brought to the hospital about a quarter past eight o'clock, on Wednesday evening, February 26th, 1817, in consequence of an injury from having run against the handle of a barrow in the dark.—I found him extremely sick, with a pulse scarcely to be felt; a fomentation was applied to his abdomen, and a glyster administered. He appeared to be in great agony. Two hours after his admission, he complained greatly of thirst and dryness of the fauces. I gave him thirty minims of tinct. opii in gruel, and ordered him to drink of barley water during the night. About four o'clock in the morning his pains seemed somewhat alleviated, and he appeared to doze. About eight o'clock the symptoms were exasperated. I directed an anodyne glyster to be given, and to be repeated occasionally. About two o'clock in the afternoon, I put him into a warm bath for a quarter of an hour, with temporary relief from pain. Soon after his removal to bed, he changed rapidly for the worse, and expired.

*Dissection.* A section of the abdominal parietes exhibited a highly inflamed state of peritonæum and intestines; coagulable lymph was found in great quantities. Effusion from the bowels must have taken place, from the circumstance of detached portions of fæces being found in the abdominal cavity. Upon separating them from each other, a transverse rupture of at least half the tube of the lesser intestine was brought into view. The whole alimentary canal was beautifully scarlet, from high inflammation. The bowels lay in moulds of lymph, and the rupture was not discernible but by separating the folds of the intestine from each other. Upon opening the stomach, fæculent matter was found in considerable quantity, intermixed with some portions of food taken since the accident.*

It is a singular fact, that rupture should have taken place in both these instances, without the least visible external mark of injury.

In the course of the last summer, a similar accident occurred in the private practice of a most respectable surgeon of this town, which terminated fatally in forty hours. Dissection discovered an extensive lesion (in a longitudinal direction) of the peritonæal coat, with an opening into the bowel large enough to admit a goose-quill, but without eversion of the inner coat. Fæces had escaped into the abdomen.

Hospital, Birmingham,
Dec. 26, 1817.

* There was eversion of the lining membrane in both of these cases.

